# Editorial: Oxidative stress link associated with mitochondrial bioenergetics: relevance in cell aging and age-related pathologies

**DOI:** 10.3389/fcell.2023.1273420

**Published:** 2023-09-05

**Authors:** Rajkumar Singh Kalra, Subodh Kumar, Dhanendra Tomar

**Affiliations:** ^1^ Immune Signal Unit, Okinawa Institute of Science and Technology Graduate University, Okinawa, Japan; ^2^ Department of Molecular and Translational Medicine, Paul L. Foster School of Medicine, Texas Tech University Health Sciences Center El Paso, El Paso, TX, United States; ^3^ Department of Internal Medicine, Section of Cardiovascular Medicine, Section of Molecular Medicine, Wake Forest University School of Medicine, Winston-Salem, NC, United States

**Keywords:** mitochondria, oxidative stress, bioenergetics, aging, age-related pathologies, neurodegenerative disease, cancer

Mitochondrial bioenergetics and its function in tissue homeostasis are vital for regulating energy processes. As cells age, mitochondrial functions are often disrupted by both intrinsic and extrinsic threats including oxidative stress (Paradies et al., 2017; Sharma et al., 2019). Although the steps involved in oxidative stress-led impaired mitochondrial bioenergetics are mechanistically conserved, involved factors/proteins may vary in different tissues and cell types (Bhatti et al., 2022; Kornicka-Garbowska et al., 2023). The regulation of these proteins in associated pathways is often compromised during aging (Paradies et al., 2010).

The original research published in this Research Topic includes two research articles on cardiomyocytes (Grün et al.; Zhang et al.) and one on human non-small cell lung cancer (NSCLC; Li et al.) along with a review article by Jagtap et al., collects new knowledge on mitochondrial function and shed light on the role of oxidative stress in altered mitochondrial bioenergetics and its relevance with aging-related pathologies. The aging-related decline in proteomic turnover, protein processing/folding, and quality control processes exaggerates the risk of oxidative stress to mitochondrial energetics (Fernandes et al., 2020; Gioran and Chondrogianni et al., 2020; Santiago et al., 2020), which potentiate progression of aging-related pathologies including cardiometabolic disorders, neurodegenerative diseases, and cancer (Dewanjee et al., 2022; Wang et al., 2022). The original research published in this Research Topic gathers new knowledge on these processes (proteomic turnover, protein processing/folding, quality control, proteotoxicity) and their oxidative stress/ROS link, impacting key cellular function in cardiomyocytes and cancer cells.

**FIGURE 1:**
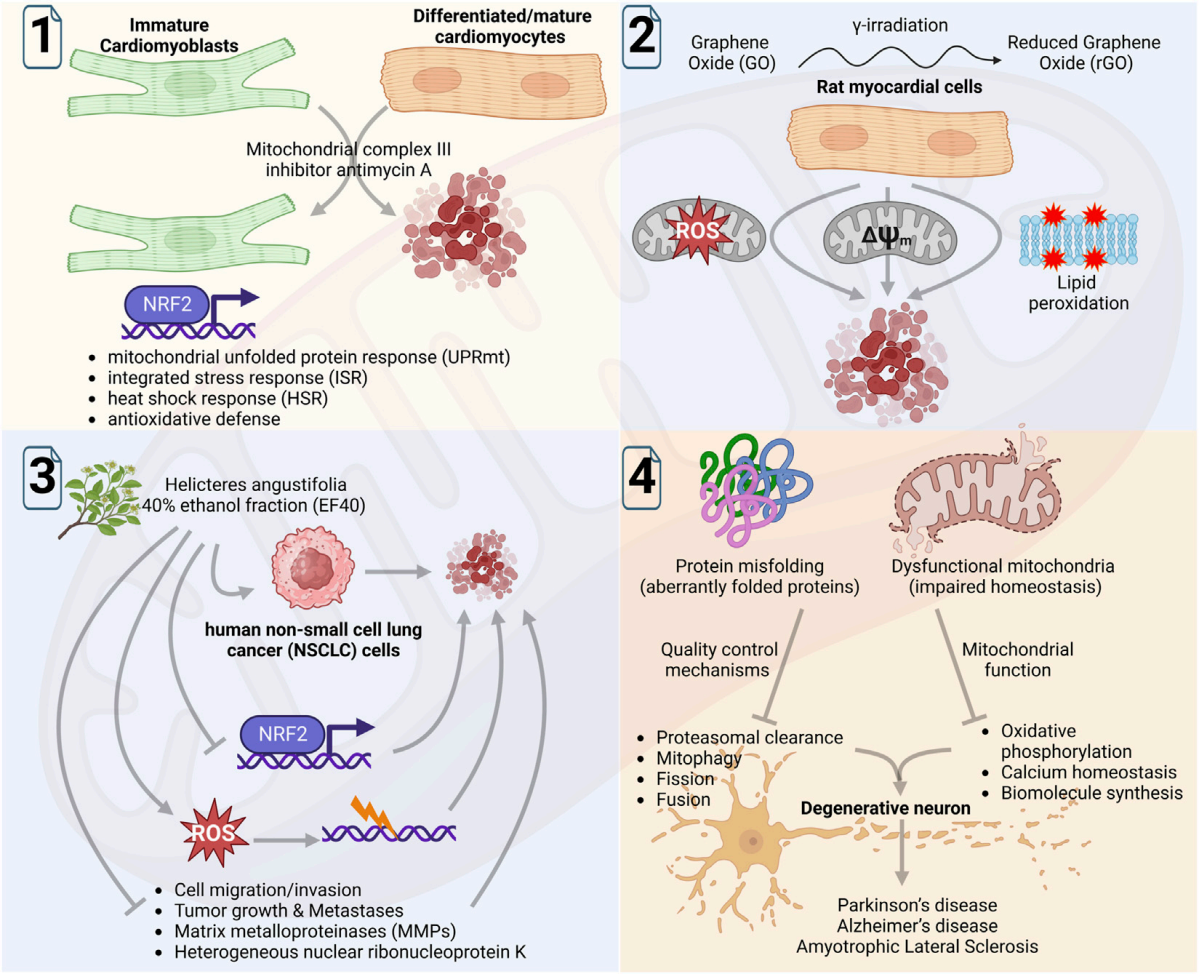
Schematic diagram showing summary of the findings by Grün et al. of mitochondrial complex III inhibitors on immature and differentiated cardiomyocytes (1); by Zhang et al. of investigating the cardiotoxic activity of graphene oxide (GO) and reduced GO (rGO) in rat myocardial cells (2); by Li et al. of analyzing the anticancer function of ROS induced by 40% ethanol fraction of Helicteres angustifolia L. in NSCLC cells (3); and by Jagtap et al., to comprehensively review the role of proteostasis in mitochondrial physiology and its function in the aging-related neurodegenerative conditions (4). The figure was Created with BioRender.com.

Grün et al. in an insightful original report, revealed that responses of immature/undifferentiated and differentiated cardiomyocytes to the inhibition of mitochondrial respiration are fundamentally different, which was reflected in their proliferation, survival, and stress responses (Figure 1; 1). Tissue homeostasis of cardiomyocytes is critical for heart development, maintenance, and function. The oxidative stress associated with ATP depletion or produced high levels of reactive oxygen species (ROS) is a feature of inhibited electron transport at the mitochondrial respiratory chain, which can be caused by selective inhibitors. Of note, while adult cardiomyocytes show high susceptibility, mouse embryonic cardiomyocytes are resistant to mitochondrial complex III inhibition. These cells were susceptible to inhibition of mitochondrial complex I, but not of complex III as no effect on cell proliferation and apoptosis was observed. In contrast, differentiated and contractile cardiomyocyte cells showed high susceptibility to complex III inhibition. In undifferentiated cells, complex III inhibition was found to induce different stress responses including unfolded protein response, heat shock response, and antioxidative defense. Interestingly, in these cells suppression of NRF2 (i.e., a transcription factor that regulates cellular antioxidant response) acquired susceptibility to complex III inhibition. These findings revealed that site specificity of the electron transport complex in the mitochondrial respiratory chain governs cell fate in immature cardiomyoblast. It reflects the fundamentally different stress tolerance and survival abilities of immature and mature cardiomyocytes, thus limited plasticity in the latter may restrict the application of cardioprotective therapies.

Zhang et al. in another original study, investigated the cardiotoxic activity of graphene oxide (GO) and reduced GO (rGO) that was found to be mediated by oxidative stress, lipid peroxidation, and mitochondrial dysfunction (Figure 1; 2). Using the rat myocardial cells, they examined the cardiotoxicity of GO and rGO (reduced by γ-irradiation and subsequently characterized by UV/visible light spectroscopy) *in vitro* and *in vivo*. The exposure of radiation absorbed dose gradually increased the rGO cytotoxicity that was linked with increasing ROS levels and declined mitochondrial membrane potential, causing mitochondrial dysfunction. Of note, mice treated with GO or rGO exhibited myocardial damage (histopathological changes in cardiac tissues) and altered activities of several enzymes/indicators associated with myocardial activity and lipid peroxidation, respectively. These data revealed evidence of GO and rGO cardiotoxicity to myocardial cells that was mediated by oxidative stress, lipid peroxidation, and mitochondrial dysfunction.

Although the oxidative stress produced by high ROS levels is central to the cardiotoxicity of myocardial cells, its lethality can be seen in cancer cells. In line with this, an original study by Li et al. provided evidence of the anticancer function of ROS, which was essentially promoted by NRF2 inhibition (Figure 1; 3). Using an *in vitro* model of human non-small cell lung cancer (NSCLC), Li et al. analyzed ethanol fractions of Helicteres angustifolia L. (*Helicteres angustifolia,* an herb used in folk medicine for its anticancer activity) root and showed that 40% ethanol fraction (EF40; consisted of several bioactive compounds) produced selective toxicity to NSCLC cells. Mechanistically, this bioactivity of EF40 was found to be linked with reduced expression of NRF2 that abundantly expresses across several cancers. Of note, EF40-led suppression of NRF2-dependent cellular defense response promoted the intracellular accumulation of ROS in the NSCLC cells. The biochemical analyses further explained the activity of EF40 in cell cycle arrest and apoptosis, promoted through the ROS-led DNA damage response. An evident decrease in matrix metalloproteinases (MMPs) and hnRNP-K expression levels in EF40-treated NSCLC cells further suggested its anti-migratory activity, which was corroborated by significantly reduced tumor growth and lung metastasis in an *in vivo* xenograft mice model. Conclusively, Li et al. provided direct evidence of the utility of ROS function in promoting anticancer activity that also reaffirmed the significance of NRF2-regulated defensive antioxidant response in cell survival.

Protein synthesis, protein processing and folding, and quality control mechanisms are critical cellular processes that are directly linked with mitochondrial homeostatic function, which progressively decline with aging. Although the role of protein homeostasis or ‘proteostasis’ in cellular function is understood, its significance in mitochondrial physiology under neurodegenerative conditions needs to be resolved. To this end, Jagtap et al. in a review report comprehensively discussed the new knowledge and developments in the stream (Figure 1; 4). The report carefully reviewed the impact of mitochondrial homeostasis disruption in different cellular processes, on other organelles, and its significance in neurodegenerative disease. It further assessed the impact of misfolded proteins on the physiological processes of mitochondria including fusion, fission, mitophagy, proteasomal clearance, and neurodegeneration. Although proteostasis enables mitochondria to recognize and respond to diverse cytotoxic stress signals that assist cells to adapt and survive, faulty processing may lead to mitochondria dysfunction and cell death. This review further underlined that the discovery of mitochondria and neurodegeneration-associated proteins is critical in understanding the disease and it could also help in designing targeted therapeutics.

Conclusively, the original research and review articles published under the present Research Topic shed light on the role of oxidative stress in altered mitochondrial bioenergetics and its outcome. It also discusses new knowledge on impaired mitochondrial bioenergetics and its relevance with age-related pathologies.
